# Inhibition of class II histone deacetylases in the spinal cord attenuates inflammatory hyperalgesia

**DOI:** 10.1186/1744-8069-6-51

**Published:** 2010-09-07

**Authors:** Guang Bai, Dong Wei, Shiping Zou, Ke Ren, Ronald Dubner

**Affiliations:** 1 Department of Neural and Pain Sciences, University of Maryland, 650 West Baltimore Street, Baltimore, Maryland 21117, USA; 2 Dental School, Program in Neuroscience, University of Maryland, 650 West Baltimore Street, Baltimore, Maryland 21117, USA

## Abstract

**Background:**

Several classes of histone deacetylases (HDACs) are expressed in the spinal cord that is a critical structure of the nociceptive pathway. HDAC-regulated histone acetylation is an important component of chromatin remodeling leading to epigenetic regulation of gene transcription. To understand the role of histone acetylation in epigenetic regulation of pathological pain, we have studied the impact of different classes of HDACs in the spinal cord on inflammatory hyperalgesia induced by complete Freund's adjuvant (CFA).

**Results:**

We intrathecally applied inhibitors specific to different classes of HDACs and evaluated their impact on inflammatory hyperalgesia. Pre-injected inhibitors targeting class I as well as II (SAHA, TSA, LAQ824) or IIa (VPA, 4-PB) HDACs significantly delayed the thermal hyperalgesia induced by unilateral CFA injection in the hindpaw. Existing hyperalgesia induced by CFA was also attenuated by the HDAC inhibitors (HDACIs). In contrast, these inhibitors did not interfere with the thermal response either in naïve animals, or on the contralateral side of inflamed animals. Interestingly, MS-275 that specifically inhibits class I HDACs failed to alter the hyperalgesia although it increased histone 3 acetylation in the spinal cord as SAHA did. Using immunoblot analysis, we further found that the levels of class IIa HDAC members (HDAC4, 5, 7, 9) in the spinal dorsal horn were upregulated following CFA injection while those of class I HDAC members (HDAC1, 2, 3) remained stable or were slightly reduced.

**Conclusions:**

Our data suggest that activity of class II HDACs in the spinal cord is critical to the induction and maintenance of inflammatory hyperalgesia induced by CFA, while activity of class I HDACs may be unnecessary. Comparison of the effects of HDACIs specific to class II and IIa as well as the expression pattern of different HDACs in the spinal cord in response to CFA suggests that the members of class IIa HDACs may be potential targets for attenuating persistent inflammatory pain.

## Background

Gene expression in the nociceptive pathway plays an important role in the induction and maintenance of persistent pain, including inflammatory pain resulting from tissue damage [1-3]. It has been found that dynamic changes in the steady-state levels of mRNAs and/or proteins in the peripheral and central nervous system occur during the development of pathological pain and that animals with specific gene knockout or knockdown exhibit altered nociceptive responses and different sensitivity to the development of pathological pain [1,4]. However, the molecular mechanisms underlying the changes of mRNA and protein levels in pathological pain conditions mostly remain unexplored except that the status of a few transcription factors, e.g., deletion of DREAM [5] and modifications of CREB [6] and NF-κB [7], were studied as single transcription factors and as a result of activated signal pathways, and mutations in a few genes have been found to be associated with the alteration of pain sensitivity in humans [8].

In addition to genetic mechanisms, gene transcription in eukaryotes is recently known to be subject to epigenetic regulation that is independent of genomic DNA sequences and is influenced largely by environmental and developmental factors [9,10]. Chromatin remodeling, DNA methylation and noncoding RNAs are three known mechanisms of epigenetic regulation [10-12]. The major force in chromatin remodeling is the modification of histone N-terminal tails [13]. One of these modifications is the acetylation of the ε-amino group of conserved lysine residues [14,15] that regulate transcription and facilitate neuronal plasticity, thus involving several neurological events [10,13,16-19]. Histone acetylation is catalyzed by histone acetyltransferase and removed by histone deacetylases (HDACs) [14,20]. The mammalian genome contains at least 18 HDAC genes that express proteins grouped into four classes: class I (HDAC1, 2, 3, and 8), class II (HDAC4, 5, 7, 9 in IIa, and HDAC6, 10 in IIb), class III (sirtuin1~7) and class VI (HDAC11) [14,20,21]. These HDAC genes are differentially expressed in the nervous system [22-24]. For example, the spinal cord expresses the genes of HDAC1~8, and 11 [25-27]. Despite the finding that no mRNA of the HDAC9 and 10 genes was detected by in situ hybridization from the spinal cord [24], microarray data deposited to the UCSC database http://www.ucsc.edu and in situ hybridization data provided on-line by Allen Institute http://www.brain-map.org showed the presence of these mRNAs and those from all seven sirtuin genes in the spinal cord. However, the roles of different classes of HDACs in pain signal transmission in the spinal cord have not been explored.

Animal studies demonstrated that the nociceptive threshold increased in adult animals who experienced stress in pre- and post-natal periods [28-31], during which the nervous system is most sensitive to environmental changes and subjected to epigenetic regulation [32]. Human studies indicated that the genetic impact on pain sensitivity in monozygotic twins diminished with increasing age that apparently accompanies more environmental exposures [33]. These observations suggest that nociceptive sensitivity may be modified by environmental and developmental factors in a way independent of genetic mechanisms. To support this notion, it was found that valproic acid (VPA) used as an antiepileptic drug for prophylactic treatment of migraine [34-37] and as an anticonvulsant to treat chronic cancer pain [38] may broadly inhibit HDACs [39-42], although VPA's effects on GABAergic activity, excitatory transmission and monoamines may affect nociception [43]. It was also found that mice expressing partial loss-of-function of HDAC4 exhibited reduced thermal nociception, but did not show a different response during the formalin test in comparison to wild-type littermates [44]. In a recent report, SAHA and MS-275 were used as HDAC inhibitors (HDACIs) after a consecutive 5-day systemic treatment and significantly reduced the second phase of the formalin test in mice [27]. Another recent report revealed that in a neuropathic pain model the neuron-restrictive silencer factor exhibits long-lasting upregulation in the dorsal root ganglion due to recruitment of histone 4 to the second promoter of the gene. Upregulated neuron-restrictive silencer factor may then suppress expression of the μ-opioid receptor and Nav1.8 genes in C-fibers [45]. Taken together, these studies suggest that epigenetic mechanisms may be involved in modification of nociception and pathological pain. However, it remains largely unknown whether the nociceptive pathway or which part of this pathway is involved. In addition, except for a differential subcellular distribution among HDACs [23,46], the potential roles of each class HDAC in the development of pathological pain are still unknown.

In the present studies, we applied HDACIs, selective to different classes of HDACs, to the spinal cord and studied modification of the inflammatory thermal hyperalgesia induced by CFA in mice. We observed that the inhibition of class II HDACs is critical to attenuate inflammatory hyperalgesia and the expression of the members in class IIa HDACs in the spinal dorsal horn was upregulated at the protein level following CFA injection. In contrast, the inhibition of class I HDAC with MS-275 showed no effect on CFA-induced thermal hyperalgesia and in addition the expression of this class of HDACs in the spinal cord was not induced by CFA.

## Results

**Figure 1:**
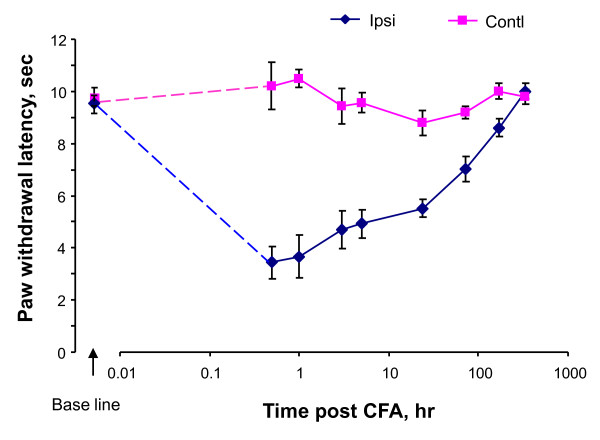
**Thermal hyperalgesia induced by CFA in mice**. Paw withdrawal latency of mice to a thermal stimulus on hindpaws were measured from both hind paws before and after unilateral CFA injection. Mean ± s.e. values from 10 animals were presented. Contl, contralateral side; Ipsi, ipsilateral side.

CFA-injected mice exhibited significant peak hypersensitivity to a noxious heat stimulus at rest 30 min after the injection (P < 0.01 compared to the baseline before the injection, Fig. 1). This typical thermal hypersensitivity appeared only on the hindpaw ipsilateral to the injection side as reported [47]. The thermal hypersensitivity was slowly resolved by 14 days after the injection. In contrast, the contralateral hindpaw showed no significant changes compared to the baseline during the tested period (P > 0.05 compared to the baseline).

**Figure 2:**
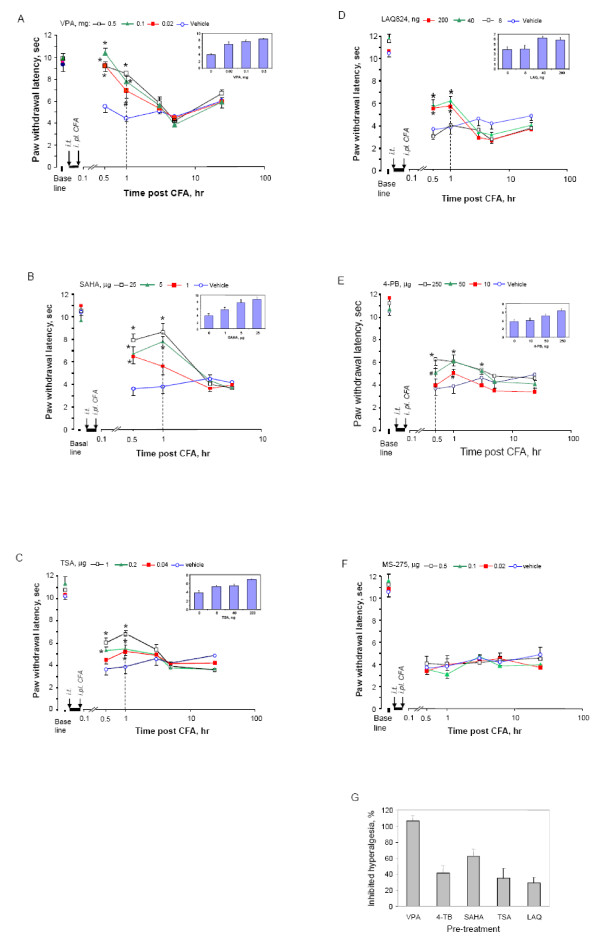
**Short-term attenuation effects of pre-injected HDACI on thermal hyperalgesia in mice**. Mice received intrathecally injected vehicle or HDACI at indicated dose 30 min before unilateral injection of CFA. Paw withdrawal latency was measured before injections as baseline, and after CFA injections as hyperalgesia response. Following HDACIs were injected for panel (in parenthesis) VPA (A), 4-BP (B), SAHA (C), TSA (D), LAQ824 (E). The inserted bar graphs showed dose dependence from the time lined up with a dash line. Mean values of eight animals per group plus standard errors are presented for the responses on the ipsilateral side. * P < 0.01, # P < 0.05: compared to vehicle at the same time point. G. Comparison of inhibition of thermal hyperalgesia by tested HDACI. Inhibition of hyperalgesia by all inhibitors at the maximal dose tested 30 min after CFA injection were calculated as described in method. One-way ANOVA analysis indicated there was no significant difference among HDAC-specific inhibitors, but all of them showed significant difference in comparison to VPA (P < 0.05).

**Figure 3:**
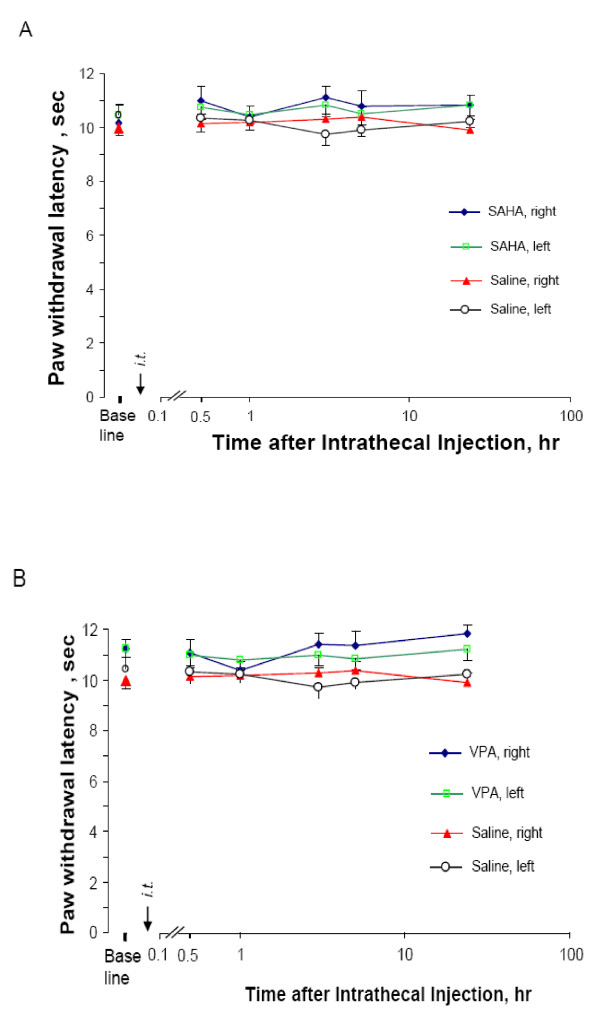
**Effects of HDACI on thermal nociception on naïve mice**. Naïve mice treated with i.t. SAHA (25 μg) or VPA (0.5 mg) were tested for their hindpaw response to a noxious heat beam for indicated time. Mean values + s.e. of PWL from 8 animals each group were presented. No significance was revealed for comparison among tested period either at the ipsilateral or contralateral side.

To explore the roles of HDACs in hyperalgesia, we applied different HDACIs to the spinal cord via intrathecal injection (i.t.). Considering that VPA was previously reported to reduce tactile allodynia in a neuropathic pain animal model after systemic administration [48] and is widely used as an HDACI to suppress class I and IIa HDACs [46,49], we first tested this inhibitor. As shown in Fig. 2A, animals receiving vehicle injection exhibited peak thermal hyperalgesia in response to CFA after 30 min as those without i.t. shown in Fig. 1. This CFA-induced hypersensitivity was largely blocked in the early time period after VPA preinjection and even showed no significant difference from the baseline. This inhibition exhibited dose dependency as shown in the inserted bar graph at the one hour time point in Fig. 2A, and declined 5 hr after CFA injection, possibly due to the clearance of the one-time injected inhibitor from tissues. However, VPA is also known to interfere with GABAergic activity, excitatory transmission and monoamines that are involved in the development of pathological pain [43]. To confirm whether HDAC activity is really involved in the inhibition of hyperalgesia, we then tested three groups of HDACIs specific to different classes of HDACs. These inhibitors are SAHA, trichostatin A (TSA), and LAQ824 to target class I and II HDACs, 4-phenylbutyrate (4-PB) to inhibit class I and IIa HDACs as well as MS-275 to block class I HDACs only [19,46,50-54]. As shown in Fig. 2, CFA-induced thermal hyperalgesia was significantly attenuated by all HDACIs, except for MS-275, in comparison to vehicle. No significant alteration of CFA-induced effect was observed for MS-275, even though a maximally soluble dose of 0.5 μg MS-275 in a 5 μl injection volume has been used. The attenuation lasted for a short period of time approximately 3 hr or less after CFA injection and showed dose dependency as indicated in the inserted bar graphs for tested dose per injection of 1~25 μg for SAHA, 0.04~1 μg for TSA, 8~200 ng for LAQ824, and 10~250 μg for 4-PB. Of these tested HDACIs, LAQ824 showed similar inhibitory effects between 40 and 200 ng suggesting saturation above 40 ng dosage. In comparison, all other HDACIs showed increasing effect following doses used. This observation prompted us to evaluate the potential of each HDACI tested by comparing their inhibitory effects on hyperalgesia at the maximal doses 30 min after CFA (Fig. 2G). With the exception of VPA, SAHA produced the strongest inhibition of hyperalgesia among tested inhibitors specific to HDAC. In another group of studies, SAHA and VPA did not interfere with thermal nociception in naïve mice within the same tested time period as shown in Fig. 3.

**Figure 4:**
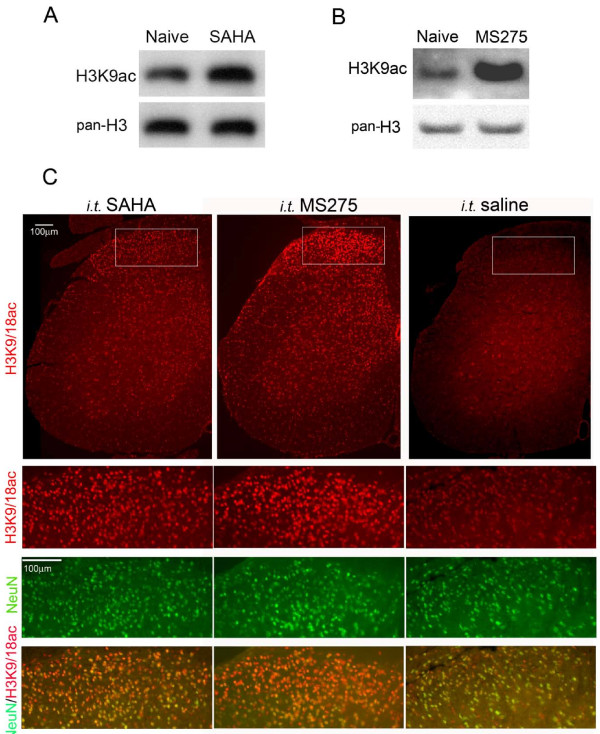
**Histone acetylation in the spinal cord after HDACI treatment**. Histone acetylation in the lumbar spinal cord of mice receiving i.t. SAHA (25 μg) or MS-275 (0.5 μg) for 30 min was analyzed by immunoblot (A, B) and immunofluorescent histochemistry (C) for antigens indicated. Animals receiving i.t. saline were used as control. Images of the H3K9/18ac signals in the left half of the lumbar spinal cord are shown in the first row in C. Immunosignals of indicated antigens in the superficial dorsal horn are presented in the rest rows in C.

We then questioned whether injected MS-275 under the condition tested executed a similar pharmacological effect as other HDACIs did. The direct targets of these inhibitors are HDACs and the functional output of inhibition of these enzymes can be assessed by the examination of the histone acetylation. Given the fact that all HDACIs tested above retain the capability to pass the blood-brain barrier [20,55-57], these inhibitors delivered intrathecally may primarily target the spinal cord and primary afferents. Therefore, we examined the effects of i.t. SAHA and MS-275 on histone 3 (H3) acetylation in the spinal cord in naïve mice. By immunoblot analysis, H3 acetylation was measured relative to total (pan-) H3 protein by an antibody specific to acetylated H3 lysine 9 residue (H3K9ac) and one to pan-H3, respectively. As shown in Fig. 4A and 4B, the relative H3K9ac signals in animals injected either with SAHA or with MS-275 were largely enhanced in comparison to that in animals receiving i.t. saline. Using an antibody specific to acetylated H3 lysine 9/18 (H3K9/18ac) for immunohistochemistry, we further observed that 30 min after the injection, the signals of H3K9/18ac robustly increased in the lumbar spinal cord (Fig. 4C). It is of interest to note that the superficial dorsal horn contained more H3K9/18ac signals. As revealed by double-labeling with NeuN, a neuronal marker, most neurons exhibited increased H3K9/18ac following SAHA or MS-275 treatments in comparison to animals receiving vehicle. These results indicate that histone acetylation in the lumbar spinal cord has been enhanced by intrathecally injected HDACIs and that MS-275 had a comparable effect on histone acetylation as SAHA.

**Figure 5:**
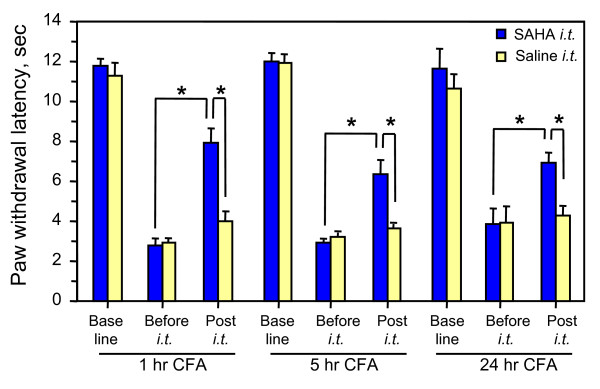
**Attenuation of existing thermal hyperalgesia by HDACI**. Mice were treated with CFA i.pl. for indicated time and then intrathecally injected with 25 μg SAHA for 30 min before measurement of thermal response. Control animals were treated with i.t. saline after CFA injection for the same time. Basal line was measured before the CFA injection. Student *t*-test was used to examine the significance as indicated groups. * P < 0.01.

The mechanisms underlying the induction of persistent pain may be different from those for its maintenance. To test whether the spinal HDAC activity could play a different role in these two events, we further studied the effect of SAHA on existing thermal hyperalgesia. SAHA was intrathecally injected in mice that had received intraplantar injection (i.pl.) of CFA for 1, 5 or 24 hr. At these time points, all animals developed peak hyperalgesia before i.t. (Fig. 5). This hypersensitivity was significantly attenuated 30 min after i.t. SAHA in all tested groups (P < 0.05) in comparison to the responses of the same animals before i.t. or to the animals receiving i.t. vehicle.

**Figure 6:**
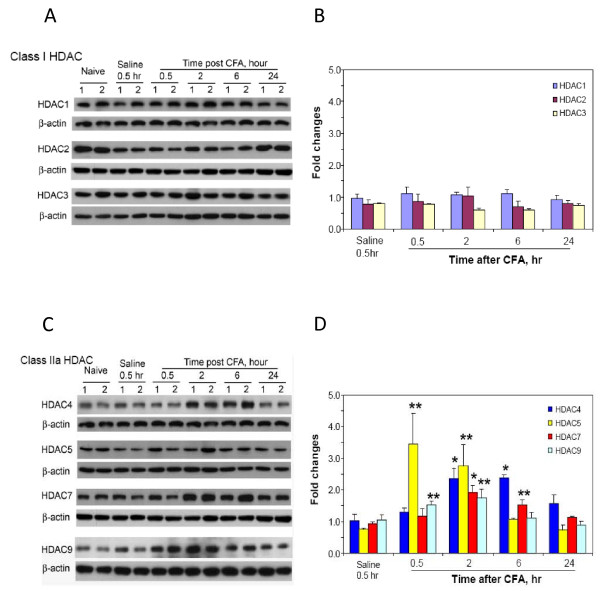
**Changes of HDACs' levels in the dorsal spinal cord after CFA injection into the hindpaw**. The levels of HDACs in the dorsal spinal cord were analyzed by immunoblot using antibodies specific to each target as indicated. Examples of blots are shown in A and C. Digitally analyzed levels of each HDAC after normalization to β-actin are shown in B for class I HDACs and in D for class IIa HDACs. Mean values plus s.e. are averaged from 4 to 6 animals. Student *t*-test was used to examine the significance as indicated groups. * P < 0.01; ** P < 0.05.

Since studies above suggest that the activity of class II HDACs in the spinal cord may be critical to induce or maintain CFA-induced hyperalgesia, it is possible that the expression of these enzymes is upregulated in response to tissue damage to support persistent pain hypersensitivity. To test this possibility, using immunoblot analysis, we quantitatively analyzed the levels of different HDACs (HDAC1, 2, 3 in class I and HDAC4, 5, 7, 9 in class IIa) in the lumbar dorsal spinal cord in animals at different time points after receiving CFA. First, we observed for each tested HDAC the bands in the sizes as suggested by manufacturers' instructions. Then, as shown by quantitative analysis in Fig. 6, the expression of members in class I HDACs was found to be stable or be slightly reduced during the time period examined, while those in class IIa HDACs were upregulated significantly to different levels. Importantly, these changes occurred in the early stage following CFA injection, but did not last longer than 24 hrs.

## Discussion

Histone acetylation has been recognized as an important mechanism in epigenetic regulation of gene transcription [14,15]. One effective approach to test whether histone acetylation impacts a biological event is to inhibit HDAC and then assess the functional output of such an inhibition. In this study, we found that pretreatment of mice with HDACIs, including TSA, SAHA or LAQ824, to target class I and II HDACs in the spinal cord significantly produced a short-term attenuation of CFA-induced thermal hyperalgesia in a dose-dependent manner. A similar reduction was induced by HDACIs specific to class I and IIa HDACs (VPA and 4-PB) [46]. However, application of MS-275 to specifically inhibit class I HDACs failed to attenuate the thermal hyperalgesia, even though it could increase H3 acetylation indicating inhibition of HDAC in the spinal cord (Fig. 4). Our results suggest that a contribution of class I HDACs to the thermal hyperalgesia induced by CFA can be excluded. Then, the inhibited class II HDACs are likely the major players in mediating the attenuation. In addition, analyses of expression of HDACs in the spinal cord following tissue damage caused by CFA revealed that the members in class IIa HDACs underwent upregulation. Therefore, our data suggest that CFA-upregulated class IIa HDACs in the spinal cord may facilitate CFA-induced thermal hyperalgesia, and that the inhibition of class IIa HDACs may be sufficient to attenuate the hyperalgesia.

In a recent report, the second phase of formalin-induced inflammatory pain was reduced by systemically injected MS-275 in the rat [27]. Specifically, MS-275 injected intraperitoneally at 3 mg/kg dosage produced even more inhibition than SAHA at 5 mg/kg dosage. In our studies, however, these two HDACIs i.t. at a similar dose difference, i.e., 0.5 μg of MS-275 vs. 1 μg of SAHA, exhibited significantly different effects on CFA-induced thermal hyperalgesia (Fig. 2). This distinct effect of MS-275 may be explained by following possibilities. First, in our studies, intrathecal injection was used to deliver HDACIs. This approach allows most injected drugs to enter the spinal cord and indeed we observed that the lumbar spinal HDACs were inhibited (Fig. 4). In contrast, systemic administration provides the opportunity for all tissues to receive administered drug and thus to participate in modification of the phenotype changes. These tissues include all structures in the nociceptive pathway from the peripheral to the central nervous system. Therefore, structures other than the spinal cord may be targeted by systemically injected MS-275 and involved in modulation of the pathological pain. Second, the inflammatory pain induced by formalin differs from that induced by CFA in aspects of duration and behavioral changes [58-60], indicating different underlying mechanisms. This notion is supported by a recent report that animals bearing a partial loss-of-function of the HDAC4 gene that belongs to class IIa HDACs exhibited reduced thermal nociception, but no changes in formalin response [44]. Third, in the present study, histone acetylation was examined only for H3K9/18 and H3K9 following MS-275 or SAHA treatment. Changes in other more than 18 lysine residues distributed among at least four different subtypes of histones, i.e., histone 2A, 2B, 3 and 4, may occur [14,15], but were not examined. Therefore, it is very likely that differential HDAC activities are involved in regulation of different models of persistent pain or that different models of persistent pain may be subjected to distinct epigenetic regulation. In support of this functional difference among HDACs, deletion of the HDAC5 gene, but not the HDAC9 gene, results in a hypersensitive response to chronic cocaine reward or stress in mice [22].

Cell-type distribution of different HDAC isoforms is another factor to be considered for the involvement of specific class HDACs in processing pain signals. Histochemically, distribution of most HDACs in the spinal cord was viewed only by mRNA in situ hybridization provided by the Allen Brain Atlas http://www.brain-map.org/. Based on this database, neurons in the spinal cord express almost all class I and II HDACs. Our data also showed that most neurons (NeuN positive) responded to HDACI treatment by exhibiting more signals of H3K9/18 and H3K9, while much fewer non-neuronal cells showed increased H3 acetylation (Fig. 4C). Taken together, these data suggest that HDAC in spinal neurons may play a major role in persistent pain.

Acetylated histones are major substrates of HDACs and thus modification of HDAC activity inevitably alters gene expression via histone-involved chromatin remodeling. Therefore, gene regulation may be considered as one molecular mechanism underlying the antihyperalgesic effect of HDACIs seen in this study. Genome-wide analyses already revealed that increases in histone acetylation by HDACI even at rest alters mRNA levels of a limited but still significant number of genes either by upregulation or by downregulation [14]. For example, infusion of MS-275 into the nucleus accumbens altered expression of ~435 genes [61]. It is known that expression of a large number of genes in nociceptive pathways impact normal nociception or persistent pain or both [1-3]. Some of those genes may potentiate hypersensitivity while some of them may attenuate hyperactivity to pain signals as evidenced by gene targeting studies in animal models [1]. Some of these genes involved in modification of nociceptive hypersensitivity may be subjected to epigenetic regulation via histone acetylation and thus mediate the antihyperalgesic effect of HDACIs. For example, recently, the expression of the metabotropic glutamate receptor 2 gene in the spinal cord and dorsal root ganglion was found to be upregulated after systemic administration of MS-275 or SAHA, and this upregulation likely mediates the inhibitory effect of these HDACIs on formalin-induced hyperalgesia [27]. Many other genes involved in persistent pain are also regulated by HDACIs. For example, HDAC inhibition increases the promoter activity of the opioid receptor genes [62-64], whose products mediate the analgesic effect of opioid peptides [65]. Another example is the brain-derived neurotrophic factor gene that has been deeply involved in the central sensitization [66,67], and importantly whose promoters and transcription are heavily regulated by histone acetylation [68-70]. Our results in the present study indicate that inhibition of class II HDACs attenuated thermal hyperalgesia, but not the normal thermal nociceptive response in naïve animals which did not have spinal HDAC induction. On the basis of all observations above, we expect that a large number of genes in the spinal cord undergo expression alteration following HDACI treatment no matter whether animals have been provoked by CFA or not, and the net effect of such expression may favor an attenuation of hypersensitivity to nociceptive stimuli, but the maintenance of normal or unprovoked nociception is not affected. We hypothesize that a pain-alleviating histone acetylation that is sensitive to class IIa HDACs may reside in the spinal cord for the development of persistent pain. The difference between the gene expression profiles resulting from the inhibition of class I HDACs and those following the inhibition of class IIa HDACs is also of interest, in view of their different effect on thermal hyperalgesia. Illustration of this difference in expression profile in the spinal cord may eventually provide insight not only of functional difference of these HDACIs, but also the molecular mechanisms underlying HDACI's antihyperalgesic activity. Our observation that alteration of histone acetylation only impacts persistent pain provides further evidence to support the notion that persistent pain is regulated by epigenetic mechanism [9].

In addition to histone, a few acetylated proteins can be the substrates of HDACs, as well [14,15,46,71] and some of these proteins may mediate the effects of HDCIs on persistent pain via gene regulation or other mechanisms. Recently, these proteins were searched globally and found to include transcription factors, proteins participating in metabolism, cell cycle and signal transduction such as NF-kappa-B-activating kinase [72-74] that involves a pathway regulating inflammatory pain hypersensitivity [7,75,76]. In view of the short duration of the HDACI effect, it is possible that acetylation in proteins other than histones was accumulated under the pressure of HDACIs, thus inducing the attenuation of hyperalgesia. This possibility is also supported by three facts. First, class IIa HDAC members are actively exported from the nucleus [14,15,77], which provides the opportunity for these enzymes to act on non-nuclear proteins that have been acetylated. Second, class IIa HDACs showed very low activity on acetylated histone used in in vitro test and were proposed to efficiently act on a narrow set of undiscovered substrates [78]. Third, class IIa HDACs interact with different non-histone proteins from those that are associated with class I or class IIb HDACs [14,79], even in face of our poor understanding of the selectivity of HDACs on acetylated lysines in histone subtypes [13].

## Conclusions

The present study demonstrates that inhibition of HDAC in the spinal cord results in a short-term attenuation of thermal hyperalgesia induced by the inflammatory agent CFA and class IIa HDAC may play a major role in this antihyperalgesic effect. This class of HDAC also exhibits upregulation in response to CFA, suggesting that the members in class IIa HDACs are potential targets for attenuation of persistent inflammatory pain. These data indicate that epigenetic regulation in the spinal cord participates in the development of persistent pain and analgesic effects resulting from inhibition of selective HDACs provides a novel target for the development of analgesic drugs.

## Methods

Animal and behavior studies. Mice (male, C57BL/6NTac, 6-7 weeks, ~20-22gr, Taconic Farm) were purchased from Taconic. All animals received care in compliance with the Guide for the Care and Use of Laboratory Animals (NIH pub No. 86-23). All experiments involving animals were conducted according to the protocol approved by the Institutional Animal Care and Use Committee at University of Maryland. Intrathecal injection was performed as reported previously [80] with a 5 μl volume to be completed in 5 min. HDACIs were obtained from the following sources (in parenthesis): VPA (Axxora Life Sciences), 4-PB (Enzo Biochem), TSA (Cell Signaling Technologies), SAHA (Cayman Chemical), MS-275 and LAQ824 (Selleck). Saline or 1% DMSO served as vehicle. Control animals received vehicle injection. After the intraplantar injection (i.pl.) of 10 μl of CFA (Sigma, diluted 1:4 with saline) to the left hindpaw, the time of paw withdrawal latency (PWL) to noxious heat was measured from both hindpaws using a system described previously [81]. The injected hindpaw showed edema and erythema indicating inflammation [81]. Intrathecal injection was completed either 30 min before CFA injection or at the selected time after CFA injection. Each experimental group included 8 to 10 animals. All experiments were performed blind for injection and behavioral test. Reversed hyperalgesia was calculated as follows. The PWL differences between the baseline and the treatment of i.t. vehicle followed by i. pl. CFA for a selected time were regarded as the 100% hypersensitivity. The PWL differences between the baseline and the treatment of i.t. tested HDACI followed by i.pl. CFA for the same selected time were regarded the modified hypersensitivity. The differences between the 100% and modified hypersensitivity are the inhibitory effect of tested HDACI.

The effect of intrathecal injection on the spinal target was examined by analyzing histone acetylation 30 min after i.t. selected HDACI by immunohistochemistry and immunoblot using three fresh mice per HDACI per technology. For HDAC expression studies, CFA or saline was injected bilaterally to naïve mice as described above for the time points indicated in Fig. 6. Four to six mice were used for each time point per treatment (CFA or saline) or for naïve animals.

Immunoblot analysis. Immunoblot analysis was carried out as described previously [82]. Naïve or treated animals were sacrificed at selected time points. Spinal cords were quickly pushed out by injecting saline into the spine. The dorsal half of the lumbar spinal cord equal to the L4 to L5 segments were quickly dissected out, frozen on dry ice and saved at -80°C until use. To test the effect of HDACI, the whole lumber spinal cord was collected. Tissue lysates were prepared by homogenizing dissected spinal cord in RIPA buffer plus protease inhibitor cocktail (Roche) in a Teflon-glass tissue grinder followed by 10 min centrifugation at 15,000xg. Lysates (16 μg protein/lane) were fractionated on 7 or 10% polyacrylamide-SDS gel in a glycine-Tris buffer and transferred to a PVDF membrane. Primary antibodies were obtained from Cell Signaling Technology for HDAC1, 2, 3, 4, 5, 7 and Santa Cruz Biotechnology for HDAC9, and were diluted by 1:1,000 in PBST. Histone was examined by an antibody specific to acetylated histone 3 Lys9 (H3K9ac, Upstate/Millipore) and by an antibody specific to pan-H3 (Upstate/Millipore). Enhanced chemical luminescence was used to reveal and quantify signals associated with the specific antigen to be detected. Signals associated with the specific immune complex were analyzed and normalized to β-actin as described previously [82].

Immunohistochemistry. Immunostaining of spinal cord was conducted as described previously [83]. Briefly, mice were perfused with 5% paraformaldehyde/PBS after selected treatments. Spinal cord was dissected out and subjected to post-fixation 5% with paraformaldehyde/PBS and cryostat protection in 30% sucrose/PBS. Transverse sections in 30 μm thick were made of the lumbar section of the spinal cord in a cryostat and mounted directly onto glass slides (Fisher Scientific). Immunostaining was performed on the glass slides with a rabbit antibody against acetylated histone 3 (H3K9/18ac, Upstate/Millipore) and a mouse monoclonal antibody against NeuN (Chemicon/Millipore). Primary antibodies were visualized by anti-rabbit IgG antibody conjugated with Cy3 and anti-mouse IgG antibody labeled with Cy2 (Jackson ImmunoResearch laboratories), respectively.

Data processing: One way ANOVA with post hoc Tukey's test was used for statistical analysis of the data among multiple groups. All data presented in figures are mean values of indicated animals plus standard error.

## List of abbreviations

CFA: complete Freund's adjuvant; HDAC: histone deacetylase; HDACI: histone deacetylase inhibitor; H3: histone 3; VPA: valproic acid; SAHA: suberoyl anilide bishydroxamide; 4-PB: 4-phenylbutyrate; TSA: trichostatin; MS-275: 2-aminobenzamides; i.t.: intrathecal injection; i.pl.: intraplantar injection.

## Competing interests

The authors declare that they have no competing interests.

## Authors' contributions

GB initiated and designed this project, analyzed data and drafted the manuscript. GB also performed immunoblot analysis and participated in animal studies for blind experiments. DW and SZ conducted animal studies and immunohistochemical experiments. KR and RD participated in experimental design, data analysis and the finalization of the manuscript. All authors have read and approved the final manuscript.
